# Assessment of Exercise Stroke Volume and Its Prediction From Oxygen Pulse in Paralympic Athletes With Locomotor Impairments: Cardiac Long-Term Adaptations Are Possible

**DOI:** 10.3389/fphys.2019.01451

**Published:** 2020-01-08

**Authors:** Marco Bernardi, Emanuele Guerra, Angelo Rodio, Donatella Dante, Vincenzo Castellano, Ilaria Peluso, Federico Schena, Yagesh Bhambhani

**Affiliations:** ^1^Department of Physiology and Pharmacology, Faculty of Pharmacy and Medicine, Sapienza University of Rome, Rome, Italy; ^2^Italian Paralympic Committee, Rome, Italy; ^3^Sports Medicine Service, Local Health Unit of Modena, Modena, Italy; ^4^Department of Human, Social and Health Sciences, University of Cassino, Cassino, Italy; ^5^Scientific Direction, Santa Lucia Foundation, Rome, Italy; ^6^Research Centre for Food and Nutrition, Council for Agricultural Research and Economics (CREA-AN), Rome, Italy; ^7^Department of Neuroscience, Biomedicine, and Movement Sciences, University of Verona, Verona, Italy; ^8^Department of Occupational Therapy, Faculty of Rehabilitation Medicine, University of Alberta, Edmonton, AB, Canada

**Keywords:** spinal cord injury, lower limb amputation, cardiac output, Paralympic sport, lower limb poliomyelitis

## Abstract

The determinants of cardiac output (CO) during exercise, i.e., stroke volume (SV) and heart rate (HR), could differ in Paralympic athletes (PAthl) with spinal cord injury (SCI) with respect to PAthl with locomotor impairments caused by different health conditions (HCs). The purposes of the present study were the comparisons of two groups of PAthl, one with SCI and the other with either amputation (AMP) or post poliomyelitis syndrome (PM), assessing the (1) peak cardiorespiratory responses and determinants (SV and HR) of CO during maximal and submaximal arm cranking exercise (ACE), respectively; (2) correlations between peak oxygen uptake (VO_2peak_) and the highest SV obtained during submaximal exercise; and (3) correlations between oxygen pulse (O_2_ pulse, ratio between VO_2_ and HR) and both SV and O_2_ arterio-venous difference [(a-v)O_2_diff]. Each athlete (19 PAthl with SCI, 9 with AMP, and 5 with PM) completed a continuous incremental cardiopulmonary ACE test to volitional fatigue to assess peak responses. In a different session, CO was indirectly measured through carbon dioxide (CO_2_) rebreathing method at sub-maximal exercise intensities approximating 30, 50, and 70% of the VO_2peak_. There were no significant differences between the PAthl groups in age, anthropometry, and VO_2peak_. However, peak HR was significantly lower, and peak O_2_ pulse was significantly higher in PAthl with AMP/PM compared to those with SCI. During sub-maximal exercise, PAthl with AMP/PM displayed significantly higher SV values (154.8 ± 17.60 ml) than PAthl with SCI (117.1 ± 24.66 ml). SV correlated significantly with VO_2peak_ in both PAthl with SCI (*R*^2^ = 0.796) and AMP/PM (*R*^2^ = 0.824). O_2_ pulse correlated significantly with SV in both PAthl with SCI (*R*^2^ = 0.888) and AMP/PM (*R*^2^ = 0.932) and in the overall sample (*R*^2^ = 0.896). No significant correlations were observed between O_2_ pulse and (a-v)O_2_diff. It was concluded that in PAthl with different HCs: (1) significant differences, as a consequence of the different HC, exist in the determinants of CO at maximal and submaximal ACE; (2) SV is a significant determinant of VO_2peak_, suggesting cardiac adaptations possible also in PAthl with SCI; and (3) SV can be predicted from O_2_ pulse measurements during submaximal exercise in both groups of PAthl.

## Introduction

Aerobic fitness levels have been well documented in recreational (Abel et al., 2008) and Paralympic Athletes (PAthl) with locomotor impairments (Baumgart et al., 2018) competing in summer (Bernardi et al., 2010) and winter sports (Bernardi et al., 2012), particularly those with spinal cord injury (SCI; Bernard et al., 2000; Bhambhani, 2002; van der Woude et al., 2002). Cross-sectional evidence indicates that trained individuals with SCI (tetraplegia and paraplegia) have significantly higher peak oxygen uptake (VO_2peak_) values compared to their sedentary counterparts (Flandrois et al., 1986; Hopman et al., 1996; Huonker et al., 1998; Schumacher et al., 2009) and that the changes in aerobic fitness in PAthl with locomotor impairments are determined by the practiced sport (Bhambhani, 2002; Bernardi et al., 2010, 2012; Baumgart et al., 2018). Possible factors that are associated with the higher VO_2peak_, i.e., the enhanced ability to transport, deliver, and utilize oxygen, are both central and peripheral in nature. Central factors, which enhance systemic oxygen availability, include significant increases in blood volume and hemoglobin concentration in endurance trained athletes with paraplegia (Schumacher et al., 2009). A primary circulatory factor that elevates their cardiac output (CO) is a significant increase in stroke volume (SV) attained in the trained state. Peripheral factors that contribute to a greater increase in maximal O_2_ extraction [i.e., (a-v)O_2diff_] include greater capillary and mitochondrial density, as well as enhanced activity of enzymes in the aerobic metabolic pathways (Bhambhani, 2002).

Previous studies have documented that SV during arm cranking exercise is significantly lower in individuals with motor complete tetraplegia (C5–C7 lesions) and complete paraplegia (T4–T11 lesions) compared to able-bodied individuals (Jacobs et al., 2002; Hostettler et al., 2012). Reduced SV was also evident in trained individuals with complete SCI between T6 and T12 compared to those who were wheelchair dependent due to permanent hip or knee injuries (Hopman et al., 1993). The primary reason for the reduced SV in individuals with SCI, particularly those with cervical and high thoracic lesions, is: (1) reduction in cardiac preload resulting from “venous blood pooling” in the abdomen and/or lower extremities, due to the absence of the skeletal muscle pump and vasoconstriction below the level of lesion (Hopman, 1994; Hopman et al., 1998); (2) reliance on relatively small upper body muscles during exercise (Theisen, 2012); and (3) reduced blood volume (Houtman et al., 2000). However, it should be noted that in individuals with a SCI above T4, blood volume significantly increases with lower limb functional electrical stimulation cycling training (Houtman et al., 2000). Comparisons among individuals with high and low paraplegia indicate that venous return from the splanchnic vasculature depends on the lesion level (Thijssen et al., 2009). Evidence also indicates that in 10 sedentary individuals with a SCI from T1 to T12, reduced venous return was the most possible cause of blunted metaboreflex-induced blood pressure and SV post exercise responses when compared to able-bodied controls (Crisafulli et al., 2009). In addition, the reduced cardiac preload, coupled with the autonomic dysfunction and sedentary lifestyle of individuals with SCI over several years, could result in chronic changes in the myocardium, thereby reducing its mass and contractility (Washburn et al., 1986; Jacobs and Nash, 2004; de Groot et al., 2006; Palmieri et al., 2010; Williams et al., 2019).

Although there are numerous studies pertaining to the metabolic and cardiorespiratory responses to exercise in athletes with SCI (Price, 2010; Theisen, 2012), research that has documented exercise SV responses in PAthl with locomotor impairments derived from lower limb amputation (AMP) and post poliomyelitis syndrome (PM) is lacking. Generally, the acute cardiorespiratory responses in the individuals with these two impairments are not physiologically altered during exercise, as the central (brainstem) control of the sympathetic innervation to their myocardium is unaffected (Birk and Pitetti, 2009). Even though the reduced muscle mass in AMP and PM could result in some decrease in venous return and therefore cardiac preload during exercise, it is unlikely that this reduction will be as high as that seen in PAthl with SCI. The greater degree of muscle atrophy resulting from chronic SCI could reduce venous return (cardiac preload) by a larger extent than that observed in PAthl with AMP and PM because of the attenuated muscle pump action during exercise.

In able-bodied individuals, the oxygen pulse (O_2_ pulse; calculated as the ratio between absolute VO_2_ and heart rate – HR) has been shown to be a valid predictor of SV during submaximal exercise (Whipp et al., 1996). Mathematically, O_2_ pulse is the product of the Fick equation variables, namely, SV and the mixed arterio-venous oxygen difference {(a-v)O_2diff_}. Whipp et al. (1996) used indwelling catheters to directly measure the (a-v)O_2diff_ during incremental cycle exercise in healthy subjects and reported a Pearson correlation of 0.80 (*p* < 0.01) between the SV calculated from the Fick equation and that predicted from O_2_ pulse. Regression equations have been developed for predicting SV during cycling exercise in untrained and trained men (Bhambhani et al., 1994; Crisafulli et al., 2007) and during upper and lower body exercise in untrained men and women (Bevegard et al., 1966; Stenberg et al., 1967; Bhambhani, 1995). While O_2_ pulse is a valid predictor of SV during exercise in healthy individuals, research that has examined the relationship between these two variables in individuals with locomotor impairments is lacking.

From an athletic performance standpoint, high levels of aerobic fitness (i.e., high VO_2peak_) were correlated with faster simulated wheelchair distance racing performance in athletes with tetraplegia (Bhambhani et al., 1995). As well, wheelchair racing performance was significantly correlated with both SV and CO in these athletes. Since SV is one of the two determinants of CO, it is important that some simple techniques for evaluating these variables during exercise be available to comprehensively monitor sport performance in athletes. Although HR can be easily recorded during exercise using wireless instruments, the measurement of SV is more challenging. Non-invasive techniques such as electrical impedance, carbon dioxide rebreathing, and acetylene rebreathing, currently used for the measurement of CO and SV, require specialized instrumentation, which may not be available in the laboratory. Furthermore, these techniques require participants to perform specific maneuvers, which may be difficult during exercise.

In light of the physiological and pathological differences between individuals with SCI and AMP/PM, comparing the maximal and submaximal cardiorespiratory responses to exercise between these two groups of PAthl is of interest to sport scientists, coaches, and athletes because they compete against each other in several classes of Paralympic sports. As well, exercise physiologists, physiatrists, cardiologists, and sports medicine physicians would find this information useful from a clinical standpoint in prescribing effective exercise programs. Therefore, the objectives of this study were to compare the: (1) peak cardiorespiratory responses and determinants (SV and HR) of CO during maximal and submaximal arm cranking exercise, respectively, in PAthl with SCI and AMP/PM; (2) magnitude of the correlation between VO_2peak_ and highest sub-maximal SV in both groups of athletes; and (3) magnitude of the correlation between the O_2_ pulse and both SV and (a-v)O_2diff_ during submaximal arm cranking exercise in these two groups of athletes. It was hypothesized that: (1) the peak cardiorespiratory responses would be significantly higher in the AMP/PM athletes compared to those with SCI; (2) the highest SV measured at sub-maximal exercise intensities would be significantly greater in athletes with AMP/PM compared to those with SCI; (3) SV would be significantly correlated with VO_2peak_ in both groups of athletes; and (4) O_2_ pulse would be significantly correlated with SV in both groups of athletes.

## Methods

### Design and Setting

All athletes completed two exercise tests on different days: an incremental graded exercise test to voluntary fatigue to determine their VO_2peak_, and three submaximal exercise tests at different intensities relative to their VO_2peak_ to assess their highest SV. Maximal data were collected in the Institute of Sport Medicine and Science of the Italian National Olympic Committee (CONI), Rome, Italy. Sub-maximal data were collected at the exercise physiology laboratory in the School of Specialty of Sports Medicine of the “Sapienza,” University of Rome (Italy). Both test sessions were carried out in accordance with the guidelines of the CONI and Italian Paralympic Committee (CIP).

### Participants

Written informed consent was obtained from two groups of elite athletes who represented Italy in the Paralympic Games over several years: 20 PAthl with SCI and 16 PAthl with lower limb impairments derived from health conditions other than SCI: AMP (*N* = 10) and PM (*N* = 6). All the testing procedures were approved by CONI and CIP for athlete testing and by the “Santa Lucia Foundation” Ethical Committee. The PAthl with SCI had complete lesions ranging from thoracic (T)4 to lumbar (L)1. Eighteen of them had a complete lesion according to the American Spinal Injury Association (ASIA) Impairment Scale (AIS) (AIS A), while two of them had an AIS C (Kirshblum et al., 2011). In all of them, the motor and the sensory levels corresponded. PAthl with SCI competed in the following Paralympic sports: three in shooting (neurological level of injury – NLI – equal to T7, T8, and T10), five in athletics (three in field events –throwing sports – one with NLI at T4 and the other two at T9 and two in track events with NLI both at T12), two in table tennis (NLI at T4 and L1, respectively), three in wheelchair fencing [two with NLI at T6 and T7, respectively, and the third one with NLI at L2 with AIS C who was excluded from the study (see the last part of the methods)], three in archery (one with NLI at T7 and the other 2 at T12), one in wheelchair tennis (NLI at T11), one in hand bike (NLI at T9), one in Alpine skiing, and one in Nordic skiing (both PAthl competing as sitting skiers, the former with NLI at T11, and the latter at L1 with AIS C). Among the PAthl with AMP (all of them with lower limb AMP), one PAthl had double thigh AMP (who was excluded from the study), three PAthl had single trans-femoral AMP, two PAthl had trans-pelvic AMP, and four PAthl had single trans-tibial AMP. The sport participation of the PAthl with AMP was as follows: one in track events (the one excluded from the study), two in field events (one in high and one in long jump), one in both wheelchair fencing and wheelchair basketball, one in cycling, one in Nordic skiing (as sitting skier), and four in Alpine skiing (all of them as standing skiers). The six athletes with PM acquired the disorder within 6 years after birth (1.92 ± 2.06, range 0.5–6 years). Three PAthl with PM had impairments in both lower limbs, while the other three had monolateral lower limb impairment. All of them were able to walk without assistive devices. They participated in the following sports: one each in archery, table tennis, shooting, Nordic skiing (as sitting skier), fencing (the athlete was excluded from the study), and field events. In summary, the subdivision of the PAthl within the sport types (Bernardi et al., 2010; Pelliccia et al., 2016) was as follows: 8 PAthl with SCI and 3 PAthl with PM competing in skill sports, 4 PAthl with SCI, 1 with PM and 6 AMP competing in power sports, 4 PAthl with SCI, 1 AMP and 1 with PM competing in intermittent sports, and 4 PAthl with SCI and 4 PAthl with AMP/PM competing in endurance sports. The pertinent age and physical characteristics of all the PAthl are summarized in Table 1 [because three PAthl were excluded from the study (see section “Statistical Analysis” at the end of the methods), they are not included in the table].

**Table 1:** Pertinent physical characteristics of Paralympic athletes (PAthl) with locomotor impairments (Mean ± SD).

Variable	SCI (*N* = 19)	AMP (*N* = 9) PM (*N* = 5) AMP/PM (*N* = 14)	Probability (P) level
Age (years)	36.8 ± 4.50	AMP 31.8 ± 7.26 PM 45.0 ± 4.24 AMP/PM 36.5 ± 9.01	SCI vs. AMP (*p* = 0.03) SCI vs. PM (*p* = 0.001) SCI vs. AMP/PM (*p* = 0.904)
Height (cm)	178.9 ± 8.59	AMP 178.5 ± 7.52 PM 168.0 ± 12.47 AMP/PM 174.1 ± 10.47	SCI vs. AMP (*p* = 0.906) SCI vs. PM (*p* = 0.032) SCI vs. AMP/PM (*p* = 0.223)
Body mass (kg)	70.7 ± 10.1	AMP 68.2 ± 9.9 PM 71.0 ± 11.31 AMP/PM 69.2 ± 10.09	SCI vs. AMP (*p* = 0.213) SCI vs. PM (*p* = 0.720) SCI vs. AMP/PM (*p* = 0.794)
Injury time period (years)	17.4 ± 7.5	AMP 16.4 ± 7.30 PM 43.2 ± 3.70 AMP/PM 25.8 ± 14.89	SCI vs. AMP (*p* = 0.127) SCI vs. PM (*p* < 0.001) SCI vs. AMP/PM (*p* = 0.04)

*SCI, PAthl with spinal cord injury; AMP/PM, PA with amputation/poliomyelitis; PM, poliomyelitis*.

### Cardiorespiratory and Metabolic Measurements

The cardiorespiratory measurements during all the exercise tests were obtained using a stationary metabolimeter (Quark b^2^, COSMED, Italy), which was interfaced with an electrocardiograph (Delta 640, Cardioline, Italy) for the incremental exercise test. The metabolimeter was calibrated according to the manufacturer’s specifications. The oxygen and carbon dioxide analyzers were calibrated using commercially available precision gases: 15% oxygen and 5% carbon dioxide, balance nitrogen. The volume turbine was calibrated using a 3 L syringe. The calibrations were verified following each test to ensure the accuracy of the data.

### Session 1: Incremental Arm Cranking Ergometer Maximal Exercise Test

The purpose of this test was to determine the VO_2peak_ of each athlete during an incremental arm cranking ergometer (ACE) maximal exercise test on an iso-power ergometer (Ergometrics 800, Ergoline GmbH, Bitz, Germany) under standardized laboratory conditions (Bernardi et al., 2010, 2012). All athletes were tested while sitting. The athletes were allowed to choose their own wheelchair or they could use the chair with a high back rest and comfortable seat provided by Ergometrics 800. Strappings were allowed, so as to replicate the specific sport conditions. The test consisted of a 3-min warm-up phase at a constant power ranging from 30 to 50 W at a cadence of 50–70 rpm, followed by increments of either 10 or 15 W every minute until voluntary fatigue; i.e., until the athletes were unable to maintain the desired cadence despite constant encouragement. In both phases, the selected power depended upon the estimated aerobic fitness and the functional classification of the athlete (Bernardi et al., 2010). These protocols were designed to complete the test in about 10 min (van der Woude et al., 2002; Bernardi et al., 2012). However, if a leveling off or decline in the VO_2_ with increasing power output was not evident at the point of fatigue, the following two criteria for VO_2peak_ (Bernardi et al., 2007) had to be attained during the exercise phase of the test: (1) HR equivalent to at least 95% (Sawka, 1986) of their age predicted maximum (220 – age, years), and (2) respiratory exchange ratio (RER) ≥ 1.10 (Bernardi et al., 2010). On line, the breath by breath data file (CPET Software Suite, Version 10.0, Cosmed, Italy) was examined to eliminate artifacts using typical cutoff for respiratory frequency, tidal volume, and oxygen and carbon dioxide expiratory fractions. Upon completion of each test, after having eliminated possible further evident artifacts, the data were averaged every seven breaths with a passing filter to smooth the curve and then around the highest identified VO_2_ values an average over 10 s was carried out to assess and quantify the VO_2peak_. The VO_2_ at each exercise stage was plotted against the power output for each athlete. From these data, ventilatory threshold (VT) and respiratory compensation point (RCP) were identified using the following Wasserman’s gas exchange criteria (Wasserman et al., 1999): lack of linearity in the relationship between carbon dioxide production (VCO_2_) and VO_2_ (i.e., non-linear increase in the RER), due to a greater increase of VCO_2_ with respect to VO_2_, and systematic decrease in end tidal CO_2_ (PetCO_2_) with concomitant increases in the ventilatory equivalent of the CO_2_ {pulmonary ventilation (VE) divided by VCO_2_}, respectively. Details of the methods used for the assessments (including other methods to validate the RCP assessment) were carried out with the previous mentioned customized software and are reported elsewhere (Patacchioli et al., 2015). From these incremental metabolic data, VO_2_ values that corresponded to 30, 50, and 70% of the VO_2peak_ were used as reference points for the CO measurements during the subsequent testing session.

### Session 2: Measurement of Cardiac Output

This submaximal test was administered on the subsequent day at approximately the same time in order to allow the participant at least 24 h rest between successive testing sessions. The CO of each athlete was determined non-invasively using the CO_2_ rebreathing technique (Collier, 1956) during seated steady-state exercise using the same ACE. The power outputs corresponding to 30, 50, and 70% of the VO_2peak_ were calculated using a formula previously assessed with a Fleish ACE and later confirmed with the same ACE used in the present study (Adami et al., 2015). The CO was measured at intensities lower than 70% of the VO_2peak_ if the preset VO_2_ value exceeded that of the RCP. This was because a physiological steady state in the PetCO_2_ would not be possible as this is an essential criterion for a valid measurement of CO using this non-invasive technique (Jones, 1988). During each stage of the test, the breath-by-breath gas exchange responses were visualized in real time, and if the data indicated that the athlete had exceeded the RCP on the basis of the criteria identified above, then the exercise intensity was lowered slightly so as to ensure that it was below the RCP. None of the athletes exceeded the RCP during the 30 and 50% exercise stages. However, at 70% VO_2peak_, four athletes exceeded the RCP, and in these cases, the work rate was reduced by approximately 5–10 W to ensure that the intensity was below the RCP.

The CO_2_ rebreathing method to assess CO is based on the Fick equation applied to CO_2_:

$${VCO}_{2}=CO\times C_{\left(v-a\right)}{CO}_{2},\mathrm{or}\;CO={VCO}_{2}/C_{\left(v-a\right)}{CO}_{2}$$

The athlete exercised for 8–9 min at each of the prescribed power outputs on the ACE before the CO assessment. The rebreathing maneuver was performed during the last 30 s of the exercise stage, while the subject was in a physiological steady state condition (i.e., no significant change in pulmonary ventilation, VO_2_, VCO_2_, and HR during the previous 2 min of exercise). In accordance with the Collier equilibrium method (Collier, 1956), the subject hyperventilated from a 3, 5, or 7 L anesthesia bag containing a mixture with CO_2_ of 9, 11, or 13% (balance oxygen), until an equilibrium was attained between the gas in the bag and the lungs. The gas concentration selected was based on the VO_2_ and PetCO_2_ criteria proposed by Jones (1988). The volume of the mixture was equal to approximately 1.5 times the tidal volume of the participant in the minute before the measurement. The PetCO_2_ tension was considered to be representative of arterial pCO_2_, while the bag CO_2_ was assumed to be indicative of mixed venous pCO_2_. The downstream correction factor proposed by Jones (1988) was utilized to correct the cardiac output values. The criterion used by the computer program for identifying pCO_2_ equilibrium was a change of less than 1 mm Hg pressure over a 5 s interval. In cases where the software was unable to detect this equilibrium point, the value was extrapolated from the line joining the points for expired PetCO_2_ at 6 to 10 s of rebreathing to that at 20 s (the software was customized to assess this point). This value has been reported to be within ±2 mm Hg of the equilibrium value. From these measurements, the SV was calculated as the ratio between CO and HR at each of the three submaximal exercise intensities. The intensity at which the highest SV was attained for each participant was then used to calculate the following variables: (1) O_2_ pulse, ml∙beat^−1^ = VO_2_/HR and (2) (a-v)O_2diff_, ml/100 ml = VO_2_/CO%. The validity of this technique in able-bodied individuals during submaximal ACE exercise has been previously established (Hopman et al., 1994). In accordance with these authors, when the steady state condition used for the CO measurement corresponded to an intensity in which RER was higher than 1, most likely between VT and RCP, a bicarbonate correction factor was adopted. An intra-class correlation of 0.85 has been reported (Myers et al., 2007) for the test-retest reliability of these measurements during submaximal ACE exercise in individuals with SCI.

### Statistical Analysis

The mean values of the pertinent physical characteristic variables in the two groups of PAthl were compared using independent “*t*” tests. The Bonferroni correction factor was used to reduce the degree of Type 1 error. One-way ANOVA was used to compare the highest SV found in the three groups of PAthl (with SCI, AMP, and PM). Two-way repeated measures ANOVA (one factor repetition) followed by all pairwise multiple comparison procedures (Student-Newman-Keuls method) was used to compare the SV values at 30, 50, and 70% of VO_2peak_ in the SCI and AMP/PM athletes. Pearson correlations were used to examine the relationships between the highest SV attained during the submaximal exercises and: (1) VO_2peak_ obtained in the ACE incremental maximal exercise test for each group of PA and (2) the corresponding values of O_2_ pulse and the latter with (a-v)O_2diff_. Linear regression analyses were used to develop regression equations for assessing if SV was a significant determinant of VO_2peak_ and for predicting SV and (a-v)O_2diff_ from O_2_ pulse in both groups of PAthl. Slopes and intercepts of the linear regression curves for SV vs. VO_2peak_ and SV vs. O_2_ pulse of the two groups of PAthl were compared using analysis of covariance (ANCOVA) to test for possible differences between PA with SCI and PA with AMP/PM. Bland-Altman analysis (Bland and Altman, 1986) was used to examine the validity of predicting SV from the calculated O_2_ pulse in both groups of PAthl. Briefly, the difference between the measured and predicted values of SV (*y* axis) was plotted against the average of these two values (*x* axis) for each individual athlete. Data points that were above or below the 95% confidence intervals (i.e., ± 2 standard deviations) were considered to be outliers. Validity between the measured SV and predicted SV was further verified using the two-way mixed effect model of the intra-class correlation coefficient (ICC). The results were considered to be significant at *α* value lower than 0.05. Data analyses were completed using SPSS computer program (Version 17.0). The results of three athletes were excluded from the statistical analysis and therefore from the study for the following reasons: (1) two fencers (one with incomplete SCI at L2 and the other with single lower limb PM) because they did not attain at least two of the three criteria previously described for a maximal cardiopulmonary exercise test and (2) one track athlete with double thigh AMP because he was declared ineligible for participation in the Paralympic Games due to cardiovascular disorders (Pelliccia et al., 2016).

## Results

### Peak Cardiorespiratory Responses

The peak cardiorespiratory responses of the two groups of athletes (PAthl with SCI vs. PAthl with AMP/PM) during the incremental ACE maximal exercise test are summarized in Table 2. In spite of a similar VO_2peak_, PAthl with SCI displayed peak HR values higher (+6.03%) than PAthl with AMP/PM (*p* = 0.015). Peak O_2_ pulse was higher in PAthl with AMP/PM than those with SCI by 17.54%. Comparisons between PAthl with AMP and those with PM (i.e., comparison within the group of PAthl without SCI) revealed no significant differences in the following peak values: power output = 151.4 ± 29.6 vs. 134.0 ± 32.1 W; VO_2_ = 2.62 ± 0.62 vs. 2.46 ± 0.59 l∙min^−1^; 38.1 ± 4.6 vs. 35.5 ± 11.0 ml∙kg^−1^∙min^−1^; HR = 179 ± 11.4 vs. 169 ± 13.4 beats∙min^−1^; O_2_ pulse = 14.8 ± 3.9 vs. 14.4 ± 2.7 ml∙beat^−1^; VE = 118.0 ± 25.6 vs. 100.4 ± 24.6 l∙min^−1^; and RER = 1.22 ± 0.10 vs. 1.19 ± 0.11.

**Table 2:** Peak exercise responses of Paralympic athletes with locomotor impairments (Mean ± SD).

Variable	SCI (*N* = 19)	AMP/PM (*N* = 9/5)	% Difference
Power output (W)	126.3 ± 41.0	145.2 ± 30.54	13.05 (*p* = 0.155)
Oxygen uptake (L/min)	2.24 ± 0.581	2.56 ± 0.593	12.59 (*p* = 0.128)
Oxygen uptake (ml/kg/min)	32.7 ± 10.90	37.2 ± 7.23	11.94 (*p* = 0.196)
Heart rate (beats/min)	186 ± 11.0	175 ± 12.6	6.03 (*p* = 0.015)
Oxygen pulse (ml/beat)	12.0 ± 3.08	14.6 ± 3.41	17.54 (*p* = 0.031)
Pulmonary ventilation (L/min)	97.1 ± 28.88	111.7 ± 25.82	13.10 (*p* = 0.143)
Respiratory exchange ratio	1.23 ± 0.079	1.22 ± 0.103	−0.80 (*p* = 0.760)

*SCI, PAthl with spinal cord injury; AMP/PM, PAthl with amputation/poliomyelitis*.

*The % difference was calculated as follows: {(AMP/PM − SCI)/AMP/PM} × 100*.

### Submaximal Cardiorespiratory Responses

The submaximal steady state intensities (%VO_2peak_) at which the CO measurements were undertaken corresponded to 36.3 ± 4.3%, 54.1 ± 6.1%, and 69.8 ± 5.8% in the PAthl with SCI and 34.9 ± 8.1%, 52.9 ± 10.1%, and 69.5 ± 13.8% in the PAthl with AMP/PM. The results of the two-way ANOVA for the SV indicated that there was significant interaction between the two factors implying that the overall trend (highest values at 70% of VO_2peak_) was similar in the SCI and AMP/PM athletes, with PAthl with SCI with lower values at each intensity. The comparison of the values at the three intensities indicated that the SV was significantly higher at 70% than 50% of VO_2peak_ and 50% higher than 30% in both groups of athletes (*p* < 0.001). The mean values of the submaximal measures for both groups are shown in Table 3. There was no significant difference between the two groups in the intensity levels at which the highest SV was reached: 68.3 ± 9.7 and 68.2 ± 9.8% VO_2peak_ in PAthl with SCI and PAthl with AMP/PM, respectively. Although CO at these intensities was not significantly different between the two groups of athletes, significant differences were observed between the groups of PAthl for SV, HR, and O_2_ pulse. SV was significantly lower in the PAthl with SCI than the PAthl with AMP/PM, but there was no difference between the PAthl with AMP (SV: 160 ± 18.2 ml) and those with PM (SV: 145.4 ± 12.78 ml). The corresponding HR was significantly higher in PAthl with SCI than those with AMP/PM. The corresponding O_2_ pulse was significantly higher in the PAthl with AMP/PM than the PAthl with SCI athletes. The CO/VO_2_ ratio and the (a-v)O_2diff_ were not significantly different between the two groups of PAthl at this submaximal exercise intensity.

**Table 3:** Cardiorespiratory and metabolic responses during submaximal exercise in Paralympic athletes with a locomotor impairment (Mean ± SD).

Variable	SCI (*N* = 19)	AMP/PM (*N* = 14)	% Difference
Power output (W)	75.6 ± 33.12	82.4 ± 21.17	8.17 (*p* = 0.512)
Oxygen uptake (L/min)	1.53 ± 0.481	1.73 ± 0.350	11.29 (*p* = 0.209)
Oxygen uptake (ml/kg/min)	22.3 ± 8.00	25.2 ± 5.20	11.61 (*p* = 0.242)
Oxygen uptake (% Peak)	68.3 ± 9.68	68.2 ± 9.98	−0.12 (*p* = 0.982)
Cardiac output (L/min)	16.7 ± 4.4	19.5 ± 3.1	14.29 (*p* = 0.051)
Cardiac output/oxygen uptake	11.1 ± 1.1	11.4 ± 0.8	2.81 (*p* = 0.361)
Heart rate (beats/min)	142 ± 15.8	126 ± 13.7	−13.19 (*p* = 0.004)
Stroke volume (ml/beat)	117.1 ± 24.7	154.8 ± 17.6	24.31 (*p* < 0.001)
Oxygen pulse (ml/beat)	10.8 ± 2.8	13.7 ± 2.3	21.63 (*p* = 0.003)
Mixed (a-v)O_2diff_ (ml/100 ml)	9.12 ± 0.89	8.82 ± 0.59	−3.40 (*p* = 0.282)

*Cardiac output was determined by carbon dioxide (CO_2_) rebreathing method at approximately 30, 50, and 70% of the VO_2peak_. The intensity at which the highest stroke volume was attained in each athlete was selected to calculate the mean value for the two athlete groups*.

*SCI, PAthl with spinal cord injury; AMP/PM, PAthl with amputation/poliomyelitis*.

*The % difference was calculated as follows: {(AMP/PM − SCI)/AMP/PM} × 100*.

### Relationship Between Peak Aerobic Power and Stroke Volume

**Figure 1:**
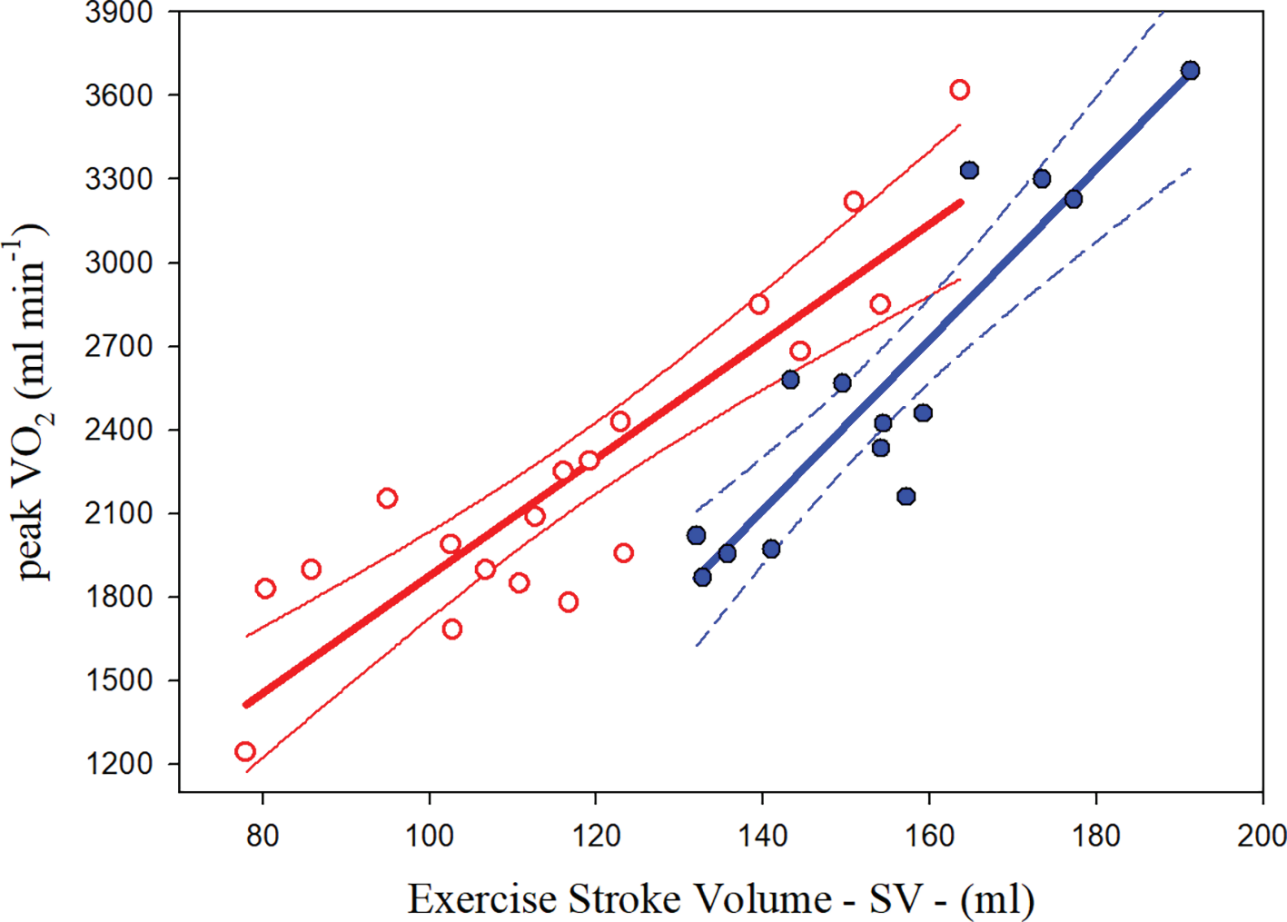
Relationships between oxygen uptake peak (VO_2peak_) and highest sub-maximal exercise stroke volume in Paralympic athletes with spinal cord injury (red scatterplot and curves) and Paralympic athletes with amputation and poliomyelitis (blue scatterplot and curves). The relative equations [Eqs. (1) and (2)] for each curve are reported in the text.

Significant correlations were observed between the VO_2peak_ assessed in the continuous maximal exercise test and the highest SV found at the steady state sub-maximal intensities in both groups of athletes. Figure 1 illustrates the scatterplots and regression curves with the respective confidence intervals (95%) for predicting VO_2peak_ from the highest SV measured at the sub-maximal intensities in each group of PA. The relative equations (Eq.) are described below:

(1) $$\begin{array}{l} \mathrm{PAthl with}\;\mathrm{SCI}:{\mathrm{VO}}_{\mathrm{2peak}}=21.03\;\mathrm{SV}-222.5;\\ \;\;\;\;\;\;\;\;\;\;\;\;\;\;\;\;\;\;\;\;\;\;\;\;\;\;\;\;\;\;\;\;\;\;\;\;\mathrm{SEE}=269.80;R^{2}=0.796 \end{array}$$

(2) $$\begin{array}{l} \mathrm{PAthl with}\;\mathrm{AMP}/\mathrm{PM}:{\mathrm{VO}}_{\mathrm{2peak}}=30.62\;\mathrm{SV}-2176.2;\\ \;\;\;\;\;\;\;\;\;\;\;\;\;\;\;\;\;\;\;\;\;\;\;\;\;\;\;\;\;\;\;\;\;\;\;\;\;\;\;\;\;\;\;\;\;\;\;\mathrm{SEE}=258.96;R^{2}=0.824 \end{array}$$

The statistical analysis showed that the two curves differed significantly for the slopes and intercepts.

### Prediction of Stroke Volume From Oxygen Pulse

**Figure 2:**
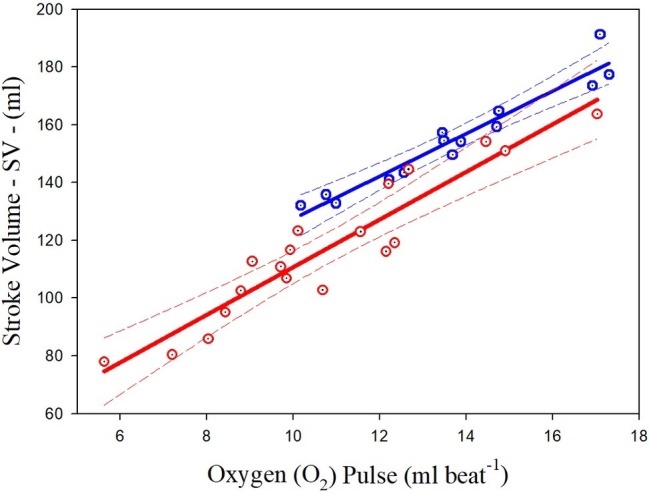
Relationships between highest sub-maximal exercise stroke volume and oxygen pulse in Paralympic athletes with spinal cord injury (red scatterplot and curves) and Paralympic athletes with amputation and poliomyelitis (blue scatterplot and curves). The relative equations [Eqs. (3) and (4)] for each curve are reported in the text.

Significant relationships were observed between the O_2_ pulse and highest SV measured at the sub-maximal intensities in both groups of athletes. The common variance of the overall data (both groups together) was 89.30%. The scatterplots and regression curves with the respective confidence intervals (95%) for predicting the SV from the O_2_ pulse values are illustrated in Figure 2 for both the PAthl with SCI (in red) and PAthl with AMP/PM (in blue). The specific equations (Eq.) are described below:

(3) $$\begin{array}{l} \mathrm{PAthl with}\;\mathrm{SCI}:\mathrm{SV}=8.26\;O_{2}\;\mathrm{pulse}+28.14;\\ \;\;\;\;\;\;\;\;\;\;\;\;\;\;\;\;\;\;\;\;\;\;\;\;\;\;\;\;\mathrm{SEE}=8.45;R^{2}=0.888 \end{array}$$

(4) $$\begin{array}{l} \mathrm{PAthl with}\;\mathrm{AMP}/\mathrm{PM}:\mathrm{SV}=7.37\;O_{2}\;\mathrm{pulse}+53.73;\\ \;\;\;\;\;\;\;\;\;\;\;\;\;\;\;\;\;\;\;\;\;\;\;\;\;\;\;\;\;\;\;\;\;\;\;\;\;\;\;\mathrm{SEE}=4.78;R^{2}=0.932 \end{array}$$

The statistical analysis showed that the two curves differed for the slopes and intercepts. Considering the whole group of PAthl, the O_2_ pulse was significantly correlated with the SV (*R*^2^ = 0.896) but not with the (a-v)O_2diff_, where the overall common variance was only 24.9%.

**Figure 3:**
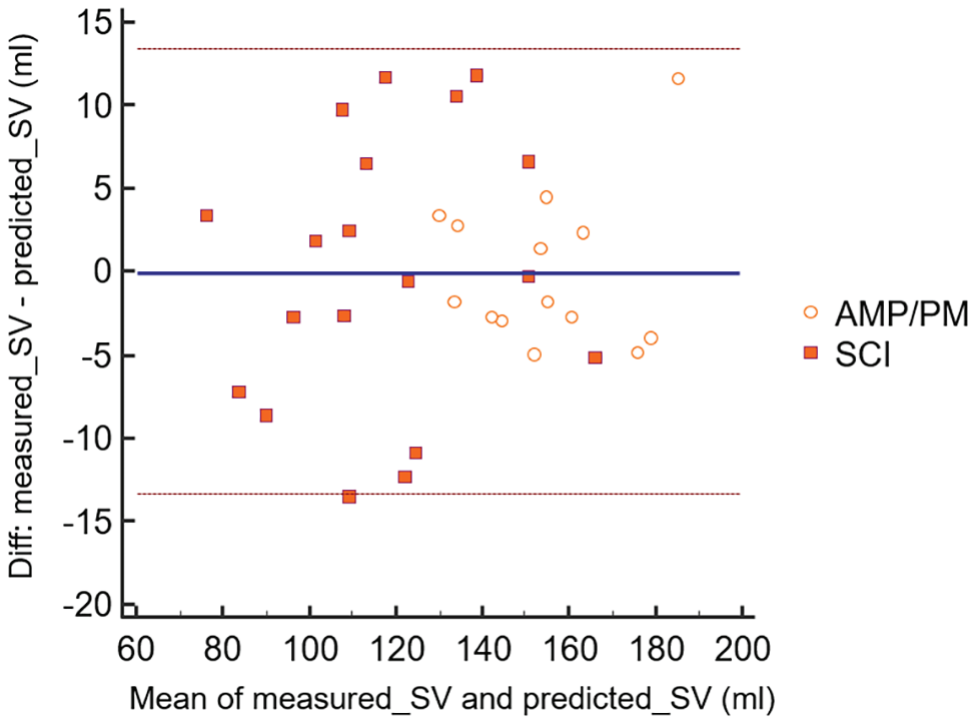
Bland-Altman analysis of measured highest sub-maximal exercise stroke volume and predicted highest sub-maximal exercise stroke volume (sv) from oxygen pulse. The scatter points are related to both Paralympic athletes with spinal cord injury (SCI) [computed with Eq. (3)] and Paralympic athletes with amputation and poliomyelitis (AMP/PM) [computed with Eq. (4)]. Mmeans, mean values of measured highest sub-maximal exercise stroke volume and predicted highest sub-maximal exercise stroke volume; Diff, difference between measured highest sub-maximal exercise stroke volume and predicted highest sub-maximal exercise stroke volume. The dashed lines indicate the 95% confidence intervals for these data points.

The Bland-Altman analysis for the PAthl with SCI and PAthl with AMP/PM is illustrated in Figure 3. It is evident that all the predicted values of SV from the O_2_ pulse measurements were within the 95% confidence intervals. Therefore, the null hypothesis that there was no proportional bias between the two measurements was accepted. These findings, in conjunction with the excellent ICC of 0.984, which had lower and upper bound confidence intervals of 0.967 and 0.992, respectively, further attested the validity of this prediction.

## Discussion

This study compared the peak cardiorespiratory responses and the CO determinants (HR and SV) during submaximal arm cranking exercise in PAthl with a thoracic or lumbar level spinal cord injury (PAthl with SCI) and PAthl with a single/double lower limb amputation or poliomyelitis (PAthl with AMP/PM). A further objective was to examine whether the SV could be predicted from the O_2_ pulse measurements in these groups of athletes. The main findings were: (1) PAthl with SCI and AMP/PM had similar values for peak aerobic fitness and CO, but the former group had significantly higher peak HR and significantly lower peak O_2_ pulse values than the latter group; (2) the highest SV measured during submaximal exercise intensities (at around 70%VO_2peak_) was significantly lower in PAthl with SCI than those with AMP/PM; (3) in both groups of PAthl, SV was a significant determinant of the VO_2peak_; and (4) SV values were highly correlated with the O_2_ pulse assessed at the same exercise intensity, implying that the latter variable could be used to predict in both groups of PAthl. The physiological basis and practical implications of these findings are discussed below.

### Peak Cardiorespiratory Responses

The VO_2peak_ of the PAthl with SCI (32.7 ml∙kg^−1^∙min^−1^) is comparable to the values reported by Hopman et al. (1993) for competitive athletes with paraplegia (30 ml∙kg^−1^∙min^−1^) during arm cranking, as well as the overall mean (31.5 ml∙kg^−1^∙min^−1^) of athletes with high and low lesion paraplegia evaluated by Bernard et al. (2000) during wheelchair ergometry. The high standard deviation (± 10.9 ml∙kg^−1^∙min^−1^) of the VO_2peak_, and therefore the wide range of VO_2peak_ values, is consistent with the fact that these PAthl competed in sports with a wide range of energy expenditure (Bernardi et al., 2010; Price, 2010) and therefore their aerobic fitness varied widely (Bernardi et al., 2012; Baumgart et al., 2018; Kouwijzer et al., 2018). Some of the lower VO_2peak_ values in the PAthl with SCI (see Figure 1) can also be explained by their lesion level. It has been reported indeed that the VO_2peak_ is inversely related to the lesion level in individuals with paraplegia (Bernard et al., 2000; Janssen et al., 2002; Nightingale et al., 2019), i.e., the higher the lesion level the lower the VO_2peak_. In the current study, the lesion level of the 19 PAthl with SCI ranged from T4 to L1. Three of these PAthl had high level paraplegia (from T4 to T6), 11 had mid-level paraplegia (from T7 to T12), and five had a low-level paraplegia (L1) with one athlete in this group having an incomplete lesion. The PAthl with high- and mid-level paraplegia had limited ability to control the trunk due to their SCI. Since trunk stabilization is important during upper body exercise (Sawka, 1986), it is likely that the performance of some of these athletes, despite using “strapping,” was compromised during the ACE, thereby resulting in a reduced CO and therefore a lower VO_2peak_. By contrast, the AMP/PM athletes were able to fully utilize their trunk musculature during ACE which enabled them to attain a higher power output and VO_2peak_.

The VO_2peak_ of athletes with AMP and PM has not been well documented. In many studies, their results have been combined with data of PAthl with other health conditions, mostly SCI, and impairments (Bernardi et al., 2010, 2012). van der Woude et al. (2002) reported a value of 35.6 ml∙kg^−1^∙min^−1^ in six elite athletes with AMP during wheelchair ergometry, but details of the lesion (unilateral vs. bilateral AMP; trans-femoral vs. trans-tibial AMP) were not provided. These values were slightly lower than those found in the PAthl with AMP in the present study (see section “Peak Cardiorespiratory Responses”). Kriz et al. (1992) reported pre-training VO_2peak_ values of 17.7 ml∙kg^−1^∙min^−1^ during arm cranking exercise in sedentary individuals with PM. These values were also considerably lower than the subgroup with PM tested in the present study. The current data on PAthl with AMP and PM fill the void that currently exists in the literature and could be useful to sport scientists, trainers, and athletes involved in Paralympic sports.

The current findings indicated that the VO_2peak_ was similar in the PAthl with AMP/PM compared to the PAthl with SCI. However, in the PAthl with SCI, these VO_2peak_ values were attained with a significantly higher HR and lower O_2_ pulse (suggesting a lower maximal SV, see discussion below) during peak exercise. This was despite the fact that in both groups of athletes, the peak HR was within their age predicted maximal HR (220 – age, ± 8 beats min^−1^), suggesting that autonomic control of the myocardium was not altered in both groups (Hopman, 1994; Bhambhani, 2002; Birk and Pitetti, 2009). The attainment of the age predicted maximal HR was also evident in the three athletes with SCI at levels between T4 and T6. This observation needs to be interpreted with caution as no autonomic assessments were done on any of the athletes who participated in this study. It is important to stress that while PAthl with AMP/PM reached the 95.7 ± 5.09% of their age predicted maximal HR, PAthl with SCI reached HR values equal to 101.7 ± 6.13%. Five PAthl with SCI (mean age of 34.2 years) showed extremely high peak HR values (range between 193 and 206 beats∙min^−1^) at the end of the maximal ACE tests (i.e., at the VO_2peak_ values). If we had not observed these extremely high HR values in these five PAthl with SCI, it is likely that there would have been no significant difference between the PAthl with SCI and with AMP/PM (Table 2). These findings suggest that this high chronotropic reserve (Palmieri et al., 2010), which allows athletes to sustain high performance with high HR and high VO_2_ values (Bernardi et al., 2010), may be a long-term central adaptation of athletes with SCI who train and compete at high intensities (Bernardi et al., 2013) over several years (see the following discussion on isokinetic circulation).

### Submaximal Cardiorespiratory Responses

Currently there is limited research that has examined the CO and SV responses during ACE exercise in PAthl. Hopman et al. (1993) measured CO by CO_2_ rebreathing (similar to this study) at 50, 70, and 80% of the peak power output during arm cranking in well-trained individuals with paraplegia. They reported no significant increase in SV between 50 and 80% of the peak power output (i.e., a plateau at approximately 50% of peak power output), a trend which was similar to that observed in untrained able-bodied individuals. In the current study, CO was evaluated at intensities between 30 and 70% of VO_2peak_, and the highest sub-maximal exercise SV was attained at 68.3 ± 9.7% in the PAthl with SCI and 68.2 ± 10.0% in the PAthl with AMP/PM (Table 3). The highest SV among the three intensities was selected even if the difference between the measurements was minimal. The higher SV at 70% of VO_2peak_ than that observed at 50% could be due to the fact that the present study included elite athletes who did considerable amount of endurance training to participate in Paralympic sport competitions. However, this cannot be confirmed on the basis of the current cross-sectional evidence from this study. Hopman et al. (1993) reported that although CO was not significantly different between well-trained individuals with paraplegia and able-bodied controls at the different exercise intensities (i.e., isokinetic circulation), the manner in which it was attained was quite different. In the able-bodied individuals, CO was attained by a lower HR and higher SV, whereas in the athletes with paraplegia, the reverse was true. This disparity between the two groups was attributed to the “venous blood pooling” in the abdomen and in the lower extremities of the individuals with higher levels of SCI, which would reduce their cardiac preload, thereby decreasing the SV. As a result, the HR had to demonstrate a compensatory increase to maintain the CO. Although Jacobs et al. (2002) confirmed that SV during submaximal arm cranking exercise at the same absolute work rate was significantly lower in untrained individuals with paraplegia and HR was significantly higher than able-bodied individuals, they reported that CO measured by bioelectrical impedance was significantly lower in the individuals with paraplegia. They claimed that the HR was unable to fully compensate for the reduced SV in individuals with paraplegia during exercise, thereby resulting in a hypokinetic circulation. These apparently contrasting results suggest that only trained individuals with SCI display the isokinetic circulation.

In the present study, able-bodied control individuals were not evaluated for comparative purposes, but the PAthl with AMP/PM could be considered a surrogate control group as their autonomic nervous system and its central (brainstem) control were intact. From another point of view, the present control group is ideal because these PAthl compete in many sports against each other. When these three variables (i.e., CO, HR, and SV) were compared between the two groups of PAthl (Table 3), there were no significant differences in CO and the CO/VO_2_ ratio between the two groups. However, the SV was significantly lower, and the HR was significantly higher in the PAthl with SCI. These physiological differences between the two groups of athletes were most likely due to the reduced muscle pump action and lack of vasomotor control below the lesion level in the PAthl with SCI. These findings support the observations of Hopman et al. (1993) discussed above and collectively suggest that notwithstanding the differences in the technique of measuring CO, well-trained individuals with paraplegia demonstrate an isokinetic circulatory pattern during submaximal and maximal arm cranking exercise. To further investigate this issue, however, the two groups should be matched for age and aerobic fitness, measuring the two variables at different exercise intensities. Additionally, measurements of cardiac dimensions need to be conducted to establish whether differences in long-term myocardial adaptations between these groups of athletes could confirm these observations.

### Relationship Between Peak Aerobic Power and Stroke Volume

This study showed that SV, measured during submaximal testing, is a significant determinant of VO_2peak_ in PAthl with SCI and AMP/PM. The common variance between these two variables was 79.6 and 82.4% in the PAthl with SCI and PAthl with AMP/PM, respectively. Previous studies (Huonker et al., 1998; Jacobs and Nash, 2004; Palmieri et al., 2010; Ordonez et al., 2013; Rosety-Rodriguez et al., 2014; Nightingale et al., 2017) have consistently demonstrated the cardiorespiratory benefits of regular upper limb aerobic exercise that accrue in untrained individuals with SCI. The present findings demonstrate that long-term central cardiac adaptation occurs in both PAthl with AMP/PM and with SCI, but to a different extent. As illustrated in Figure 1, PAthl with AMP/PM tend to have SV values higher than those with SCI of about 30 ml at low levels of VO_2peak_ (less fit athletes) and about 15 ml at high VO_2peak_ levels (most trained athletes). This greater long-term adaptation in PAthl with AMP/PM is probably due to their ability to walk and, in some cases, participation in a standing sport. The reduced SV observed in the PAthl with SCI compared to those with AMP/PM during exercise was most likely due to a reduction in their cardiac preload as a result of chronic venous blood pooling in the lower extremities (Hopman, 1994; Hopman et al., 1998; Jacobs et al., 2002; Theisen, 2012) and reduction in blood volume (Kinzer and Convertino, 1989; Houtman et al., 2000). These data and the related equations show that PAthl with SCI rely on peripheral adaptations to exercise to increase their VO_2peak_ to a greater extent than PAthl without SCI. Reduced cardiac preload as well as several years of reduced physical activity could induce chronic changes in the myocardium resulting in reduced cardiac wall thickness and/or heart cavity dimensions, thereby influencing the myocardial contractility and SV of these athletes (Washburn et al., 1986; Palmieri et al., 2010; Williams et al., 2019). Animal research has indicated that the cardiac atrophy is dependent on the severity of the SCI (Squair et al., 2018). In this study, no echocardiographic measurements were undertaken to study heart dimensions, and therefore, it is difficult to identify whether these factors could account for the differences in SV between the two groups of PAthl. Further research is needed to elucidate the myocardial changes that occur from long-term aerobic detraining and training and their effect on cardiovascular dynamics and aerobic fitness in PAthl. However, the current physiological assessment has demonstrated an important clinical use of the relationship between highest SV at submaximal exercise intensities and VO_2peak_. One athlete with double thigh AMP was excluded from the study because, few years following this assessment, he was declared ineligible for Paralympic sport due to dilated cardiomyopathy (Pelliccia et al., 2016). This athlete was definitely an outlier in this relationship, because his SV was more than two standard deviations lower than that predicted for his aerobic fitness. His relatively high VO_2peak_ was mainly due to peripheral adaptations to exercise. However, his predicted SV from the O_2_ pulse measurement (data not shown in the present paper) was fairly accurate (see next section of the paper).

### Prediction of Stroke Volume From Oxygen Pulse

The current findings supported our hypothesis that the O_2_ pulse would be a valid predictor of SV during arm cranking exercise in the PAthl with SCI and AMP/PM [Eqs. (3) and (4) and Figure 2]. These findings corroborate previous reports on healthy, able-bodied individuals, which have shown that O_2_ pulse is a valid predictor of SV during arm cranking (Bevegard et al., 1966; Stenberg et al., 1967; Bhambhani, 1995) and leg cycling (Bhambhani et al., 1994; Whipp et al., 1996). The common variance (*R*^2^) between these two variables (Stenberg et al., 1967; Bhambhani, 1995) using the CO_2_ rebreathing technique (73%) was lower than those found in the present study for PAthl with SCI and AMP/PM. The current findings demonstrate that the common variance between the O_2_ pulse and SV was 88.8 and 93.2% in the SCI and AMP/PM athlete groups, respectively. This was despite the differences in: (1) the determinants of their CO during submaximal exercise; (2) the reduced muscle mass available for recruitment in the athletes with AMP/PM; and (3) impaired ability to recruit musculature below the lesion level in the PAthl with SCI. The latter two points would increase the degree of venous blood pooling, which would normally occur during seated upper body exercise (Sawka, 1986), thereby affecting differently the cardiac preload and the SV. The Bland-Altman analysis in Figure 3 indicated that all data points were within the 95% limits of agreement in both the athlete groups, supporting the validity of these predictive equations. Two additional points should be noted: (1) because SV remains fairly constant between 50 and 80% of peak power output during arm cranking exercise (Hopman et al., 1993), these regression equations enable sport scientists to predict the SV within this exercise range without the need for additional tests, and (2) since O_2_ pulse was not significantly correlated with the (a-v)O_2diff_ during submaximal arm exercise (the Bland-Altman analysis failed), it is likely that the changes in O_2_ pulse, which occur with training primarily, reflect modifications in the SV (Bhambhani, 1995). However, this hypothesis needs to be further investigated with appropriately designed research studies.

## Limitations of the Study

The following limitations should be considered when interpreting the results of this study. First, all the athletes were tested during arm cranking and not the exercise mode that was specific to their competitive sport. It is possible that this could have influenced the cardiorespiratory responses to some extent. However, if we had chosen to test the athletes during wheelchair ergometry, an exercise mode that is routinely used to test athletes with locomotor impairments, this could have biased the results of the athletes who used wheelchairs for training and competition; e.g., track and tennis athletes in the SCI and the AMP/PM groups (Abel et al., 2008). Second, the PAthl with AMP/PM group consisted of a small number of athletes with each of these disorders and therefore different impairments. However, we felt justified in combining these two impairments because neither of them alters autonomic function, which was very important to control for this type of exercise study. Third, there was some heterogeneity in the PAthl with AMP who participated in this study. Five of them had single trans-femoral amputation and four had single trans-tibial amputation. Nevertheless, it is unlikely that differences in muscle mass of the lower extremities would have influenced their performance during arm cranking exercise, because all these athletes had complete control of their trunk and upper body musculature. Finally, there were significant differences in the age of the athletes with SCI compared to the two groups of athletes with AMP and PM, which could influence the cardiovascular responses during exercise. The SCI Athletes were significantly older than the athletes with AMP by 4.3 years and significantly younger than the athletes with PM by 8.2 years. However, when the ages of the athletes with AMP and PM were pooled, there was no significant difference between the athletes with SCI and AMP/PM. It should be noted that both groups of athletes attained their age predicted maximum heart rate (220 – age), as indicated in Table 2, and the mean HR value was significantly different between the two groups of athletes. Furthermore, the peak cardiorespiratory responses of the athletes with AMP and PM were not significantly different (see section “Results”) despite a significant difference in their age, suggesting that the differences between the athletes with SCI and AMP/PM were most likely due to their health conditions. Despite these limitations, the ability to predict SV from the O_2_ pulse was robust in both the groups of athletes.

## Conclusions

In two groups of elite PAthl with different locomotor impairments, but with similar aerobic fitness (VO_2peak_), the cardiac output determinants were significantly different. PAthl with SCI displayed significantly higher HR and lower O_2_ pulse values during peak arm cranking exercise than PAthl with AMP/PM. The ability to reach high HR values in PAthl with SCI who had no neural disruption to the myocardium was most likely due to their chronic aerobic training. The highest SV found at submaximal intensities (at around 70% VO_2peak_) in PAthl with SCI was significantly lower than that measured in PAthl with AMP/PM. The corresponding HR values were significantly lower in PAthl with AMP/PM than those with SCI. When assessing training status of PAthl, HR values should be carefully considered in conjunction with their specific impairment. In both groups of athletes, SV was significantly correlated with VO_2peak_. The O_2_ pulse was a valid predictor of SV in both the athlete groups and was not significantly correlated with the mixed (a-v)O_2diff_. O_2_ pulse is a useful physiological variable to evaluate changes in SV in PAthl when direct measurement is not feasible. Further research is needed to examine whether the changes in SV resulting from long-term aerobic training can be tracked by evaluating the changes in O_2_ pulse in these athletes.

## Data Availability Statement

The datasets for this article are not publicly available because they are related to a private database. Requests to access the datasets should be directed to marco.bernardi@uniroma1.it.

## Ethics Statement

This study was carried out in accordance with the recommendations of the Italian National Health service. All the testing procedures were approved by the Italian National Olympic Committee and Italian Paralympic Committee for athlete testing and by the “Santa Lucia Foundation” Ethical Committee. Written informed consent was obtained from all subjects. All participants gave written informed consent in accordance with the Declaration of Helsinki.

## Author Contributions

MB, VC, FS, and YB contributed to conception and design of the work. MB, EG, and AR participated in data acquisition. MB, EG, AR, DD, IP, FS, and YB analyzed and interpreted the data. MB, EG, and VC contributed to recruitment of athletes. MB and VC contributed to clinical and functional classification (scales) of the subjects. MB, EG, DD, FS, and YB drafted the manuscript. MB, AR, IP, and YB revised the manuscript. MB and EG contributed to clinical evaluation of subjects. DD and IP performed the statistics of the work. MB, EG, AR, DD, VC, IP, FS, and YB approved the final version of the manuscript.

### Conflict of Interest

The authors declare that the research was conducted in the absence of any commercial or financial relationships that could be construed as a potential conflict of interest.
